# Optical Frequency Metrology of an Iodine-Stabilized He-Ne Laser Using the Frequency Comb of a Quantum-Interference-Stabilized Mode-Locked Laser

**DOI:** 10.6028/jres.112.022

**Published:** 2007-12-01

**Authors:** Ryan P. Smith, Peter A. Roos, Jared K. Wahlstrand, Jessica A. Pipis, Maria Belmonte Rivas, Steven T. Cundiff

**Affiliations:** JILA, University of Colorado and National Institute of Standards and Technology, Boulder, CO 80309-0440; Department of Physics, University of Colorado, Boulder, CO 80309-0390; JILA, University of Colorado and National Institute of Standards and Technology, Boulder, CO 80309-0440; Department of Aerospace Engineering, University of Colorado, Boulder, CO 80309-0429; JILA, University of Colorado and National Institute of Standards and Technology, Boulder, CO 80309-0440

**Keywords:** femtosecond (fs) lasers, laser frequency control, optical clocks, optical frequency comb, optical frequency measurement, stabilized lasers

## Abstract

We perform optical frequency metrology of an iodine-stabilized He-Ne laser using a mode-locked Ti:sapphire laser frequency comb that is stabilized using quantum interference of photocurrents in a semiconductor. Using this technique, we demonstrate carrier-envelope offset frequency fluctuations of less than 5 mHz using a 1 s gate time. With the resulting stable frequency comb, we measure the optical frequency of the iodine transition [^127^I_2_ R(127) 11-5 i component] to be 473 612 214 712.96 ± 0.66 kHz, well within the uncertainty of the CIPM recommended value. The stability of the quantum interference technique is high enough such that it does not limit the measurements.

## 1. Introduction

Absolute optical frequency metrology is the measurement of an optical frequency referenced to the primary frequency standard, a cesium atomic clock [1]. This microwave frequency standard differs from optical frequencies by more than five orders of magnitude, making direct comparison impossible. In the past, this problem has been overcome by means of arduous frequency chains [2]. More recently, the introduction of the frequency comb produced by femtosecond mode-locked lasers [3–6] has dramatically simplified the link between optical and microwave frequencies, and frequency combs have become the predominant tool used for optical frequency metrology. The frequency comb is a necessary technique for use in optical atomic clocks. Optical frequency standards now surpass cesium reference standards in stability and rival or potentially surpass them in accuracy [2,7,8].

A mode-locked laser produces a wide comb of evenly spaced optical frequency modes in the frequency domain [9]. The frequency comb may be seen as a direct result of a sequence of coherent short pulses in the time domain. The optical frequency of a given comb line is *ν_n_* = *n f*_rep_ + *f*_0_, where *n* is an integer comb line index, *f*_rep_ is the repetition rate (spacing between comb modes), and *f*_0_ is the carrier-envelope offset frequency, the rate at which the carrier-envelope phase slips from pulse to pulse due to the difference in the group and phase velocities within the laser cavity. When these two RF frequencies are referenced to the primary cesium standard, the absolute optical frequencies of all the comb lines are known. To precisely determine the unknown frequency of a continuous-wave (CW) laser, one must first measure the frequency (by a standard method such as a wavemeter) to within the repetition rate of the laser. Then one can simply measure the heterodyne beat *f*_beat_ between the CW laser and the closest line in the frequency comb. This RF beat results from the interference of these two optical frequencies. The unknown frequency of the laser is then

$$\nu_{\text{unknown}}=n\times f_{\text{rep}}+f_{0}\pm f_{\text{beat}}.$$ (1)

Stabilizing the frequency comb is helpful for simplifying optical metrology measurements. Stabilization is accomplished by comparing the two parameters of the comb, *f*_rep_ and *f*_0_, to a stable reference. Typically, stabilization of *f*_rep_ is straightforward, performed by detecting the rate of pulses onto a photodetector, and then feeding back to an actuator that controls the cavity length of the mode-locked laser. However, detection and stabilization of *f*_0_ is more involved, and several techniques have been developed for this purpose. In one common technique to measure the carrier-envelope offset frequency, known as *ν* - to - 2*ν* self-referencing, a frequency-doubled comb line from the low-frequency wing of the spectrum of the mode-locked laser, after having been broadened to span an octave, is optically interfered with a comb line that is nearly twice its frequency in the upper wing of the spectrum. The heterodyne beat frequency between these two lines is *f*_0_ [3]. In this work, we demonstrate the applicability to optical frequency metrology of a recently developed alternative self-referencing technique that uses quantum interference control of photocurrents in semiconductors [10–14]. This allows the two-color interferometer of the standard technique to be replaced by a semiconductor device.

Quantum interference control (QIC) of injected photocurrents in semiconductors exploits phase-sensitive interference between one- and two-photon absorption pathways from the valence to the conduction band [10,11]. Two-photon absorption of light at frequency *ν* (below the band gap) and one-photon absorption of 2*ν* (above gap) light separately produce carrier populations that are symmetric in *k*-space, resulting in no net current flow. However, when both absorption pathways exist simultaneously, quantum interference between them can occur, creating an asymmetry in *k*-space for the carrier population and therefore a net current in the semiconductor. The current injected in the semiconductor due to this interference depends sinusoidally on the phase difference of the two absorption pathways, 2*ϕ_ν_* − *ϕ*_2_*_ν_*, where *ϕ_ν_* and *ϕ*_2_*_ν_* are the optical phases of the two spectral components involved. Light pulses containing optical frequencies that differ by a factor of two (an octave-spanning spectrum) can excite both onezand two-photon absorption simultaneously. The carrier-envelope phase is, up to a constant offset, proportional to 2*ϕ_ν_* − *ϕ*_2_*_ν_*, and thus the injected current due to quantum interference oscillates at *f*_0_ [11]. Thus, using QIC we are able to replace the doubling crystal and optical interferometer of the standard self-referencing apparatus with two-photon absorption and quantum interference in a semiconductor. The resulting measurement has a high enough signal-to-noise ratio to be used to stabilize *f*_0_ [14], and here we apply this technique of measuring and stabilizing *f*_0_ to optical frequency metrology.

## 2. Experiment

**Fig. 1:**
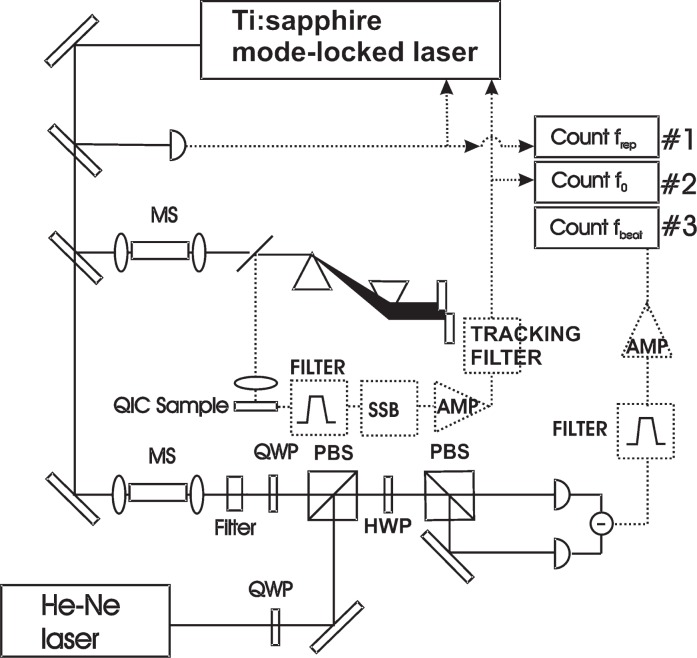
Experimental setup for optical metrology using quantum interference control (QIC). The QIC setup stabilizes *f*_0_ and also provides a countable signal. MS, microstructure fiber; QWP, quarter wave plate; HWP, half wave plate; PBS, polarizing beam splitting cube; AMP, RF Amplifier; TF, Tracking Filter; FILTER, bandpass filter; SSB, single side-band mixer.

To demonstrate optical frequency metrology we chose to measure the frequency of an iodine-stabilized He-Ne laser. The absolute frequency of the He-Ne laser is determined by simultaneously measuring the three frequency parameters on the right side of Eq. (1). In addition, the index *n* of the comb line that is used in the heterodyne detection with the He-Ne laser and the sign of the *f*_beat_ are determined by prior knowledge of the He-Ne laser frequency. The experimental setup is shown in Fig. 1. The laser being measured is a commercial iodine-stabilized He-Ne laser,^1^ which uses a saturation spectroscopy technique to lock the lasing frequency to a specific hyperfine component of molecular iodine vapor in a temperature-stabilized cell. The He-Ne laser is locked to the ^127^I_2_ R (127) 11-5 i transition. A rough measurement of the He-Ne laser frequency using a commercial wavemeter provides enough information to determine the closest comb line index. The vapor iodine cell of the He-Ne laser is frequency calibrated by the Bureau International des Poids et Mesures (BIPM) within 12 kHz [15].

As mentioned above, stabilization of the comb is required for use in optical frequency metrology. We phase lock *f*_rep_ of a mode-locked Ti:sapphire laser to a synthesized signal near 94 MHz by controlling the cavity length of the laser with a piezoelectric transducer. The pulse train is directly measured with a photo-diode, and *f*_rep_ is counted (counter 1 in Fig. 1). The synthesizer and all counters are referenced to a commercial Cs atomic clock.^2^ A portion of the output of the laser is spectrally broadened in a microstructure fiber and then sent through a prism pair, which compensates for delays between the spectral components of *ν* and 2*ν* due to dispersion in the fiber. This light is directed onto the QIC semiconductor device, which is low-temperature grown GaAs with AuGe electrodes, separated by about 30 μm, deposited on the surface [13]. The carrier-envelope offset frequency (*f*_0_) signal, derived from the QIC current injected across the electrodes and then amplified using a transimpedence amplifier, is typically detected near 400 kHz. It is necessary to detect *f*_0_ at such a low frequency because the intrinsic capacitance of the sample structure acts as a low-pass filter [16]. The signal is then shifted to near 82 MHz (using a second synthesizer phase locked to the first) using a single-sideband mixer [12] in order to match the design frequency of the stabilization electronics. An RF tracking filter is used to improve the *f*_0_ signal-to-noise ratio (originally ~20 dB with a 10 kHz resolution bandwidth) in order to make the measurement of this parameter more robust. The tracking filter phase locks a voltage controlled oscillator (VCO) to the up-shifted *f*_0_. The VCO output from the tracking filter is then mixed with a synthesized signal at 7/8 *f*_rep_ in order to generate an error signal for phase locking of *f*_0_ by tilting the cavity end mirror [17]. We count the output from the tracking filter (counter 2 in Fig. 1) as part of the unknown optical frequency determination via Eq. (1). Although introducing the tracking filter had the potential to add phase noise to the measurement, we find that the *f*_0_ noise contributions are insignificant (five orders of magnitude smaller) compared to other sources of noise in the final determination of the unknown optical frequency. In addition, previous *f*_0_ phase noise measurements using the tracking filter show comparable noise performance to those without a tracking filter (data not shown). For comparison, we also made measurements using the standard *ν* - to - 2*ν* self-referencing technique.

A heterodyne beat between the He-Ne laser and the frequency comb provides the final parameter necessary in Eq. (1) for determining the He-Ne frequency. Light from the mode-locked laser, spectrally broadened using a microstructure fiber, is combined with the output of the He-Ne laser using a pair of polarizing beam splitters. We detect the heterodyne beat between the He-Ne and the nearest comb line [*f*_beat_ in Eq. (1)] using two photodiodes in a resonant circuit. The resonant circuit enhances the signal-to-noise ratio at the resonant frequency of 65 MHz. The heterodyne detection scheme was designed for each detector to measure *f*_beat_ 180° out of phase, such that a subtraction of the two signals adds the desired signal while suppressing amplitude fluctuations that are common to both arms. The combination of this balanced detection and resonant enhancement in the detectors increased the signal-to-noise ratio by approximately 5 dB to more than 25 dB, which was critical for accurate counting. The beat signal was then filtered, amplified, and sent to a third frequency counter (counter 3 in Fig. 1). We determine the frequency of the He-Ne laser by adding the recorded RF frequencies via Eq. (1) after subtracting the frequency shift of the single side-band mixer.

For a valid comparison of our measurement to the International Committee for Weights and Measures (CIPM) recommended value [18], the proper operating parameters must be used. All conditions are within the range of recommended values except for the intra-cavity optical power of our He-Ne laser. The CIPM recommends specific operating conditions for the iodine-stabilized He-Ne; recommended is a modulation depth of 6 ± 0.3 MHz, a cold finger temperature of 15 ± 0.2 °C, and a cell wall temperature of 25 ± 5 °C, and an intracavity optical power of 10 ± 5 mW. Our commercial He-Ne laser operates with 6.0 ± 0.3 MHz modulation depth, a cold-finger temperature of 15.04 °C, a cell wall temperature of 28 ± 2 °C, and an output power of 128 μW. Using the manufacturer estimated output coupler transmission of 0.7 %, our intracavity optical power, calculated to be 18.3 mW, is nearly twice the CIPM recommended value of 10 mW. We require the higher power to ensure sufficient signal-to-noise of the beat between the He-Ne and a comb line. Though this intracavity power is outside the recommended range, we can correct for the frequency shift in the He-Ne laser that this causes based on empirical data from the manufacturer.

We test for the possibility of systematic errors in the detection of the He-Ne/comb beat (*f*_beat_) by altering the repetition rate to shift the comb line of interest to the opposite side of the He-Ne laser line and making another measurement. Because this reverses the sign of the He-Ne/comb beat in Eq. (1), systematic errors in the measurement of this beat would be evident in the resultant optical frequency measurements. This technique establishes an upper limit on the systematic error of 1.84 kHz for each data set for the measurement of the He-Ne/comb beat.

## 3. Results and Analysis

**Fig. 2:**
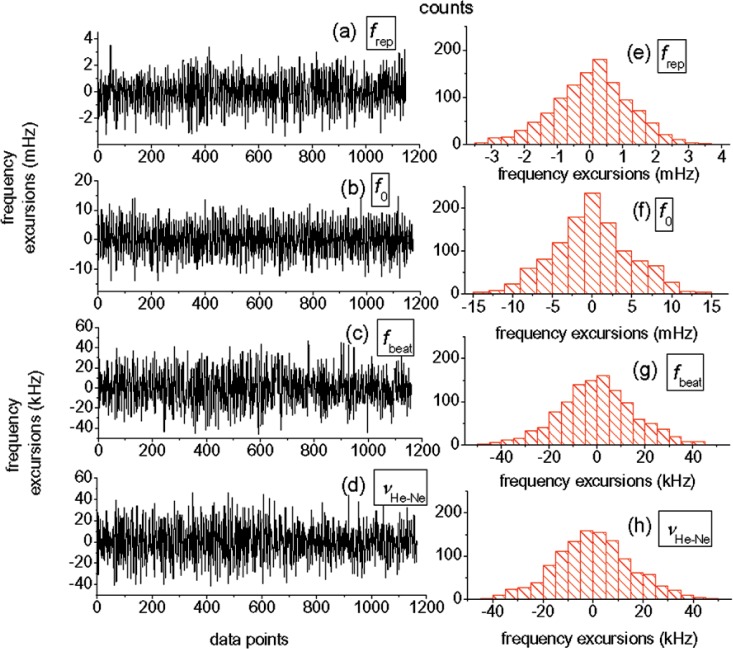
Data from the longest continuous run. Outliers caused by cycle slips in one of the phase-lock loops are removed. Plots a–d show the frequency stability for the repetition rate, the carrier-envelope offset frequency, the beat frequency, and the calculated He-Ne laser frequency, respectively. Plots e–h are histograms for these frequencies.

We first analyze the stability of each of the three measured RF frequencies independently. The repetition rate (*f*_rep_), the carrier-envelope offset frequency (*f*_0_), and the He-Ne/comb beat frequency (*f*_beat_) exhibited standard deviations for a data set of ~1000 points (using a 1 second gate time) of 1.7 mHz (Δ*f*_rep_/*f*_rep_ = 1.2 × 10^−11^), 4.8 mHz (Δ*f*_0_/*f*_0_ = 1.1 × 10^−8^), and 15.1 kHz (Δ*f*_beat_/*f*_beat_ = 2.3 × 10^−4^), respectively (see Fig. 2(a–c)). The stabilities of and correlations between the three frequencies measured yield the fluctuations in the measured He-Ne frequency shown in Fig. 2(d). In order to lower the uncertainty in our measurement of the He-Ne frequency, we averaged the results from several measurements for a total of 2600 data points taken over the course of one day.

We estimate the magnitudes of the sources of error in the measurement of the He-Ne frequency to ensure that we have properly considered relevant contributions and to determine the relative contribution to the error due to the new technique of measuring *f*_0_. The fluctuations in *ν*_He-Ne_ can be found using an error propagation analysis [19] and Eq. (1) to be

$$\begin{array}{l} \sigma_{\nu_{He-Ne}}^{2}=n^{2}\sigma_{f_{\text{rep}}}^{2}+\sigma_{f_{\text{beat}}}^{2}+\sigma_{f_{0}}^{2}\pm 2r_{f_{\text{rep}}f_{\text{beat}}}n\sigma_{f_{\text{rep}}}\sigma_{f_{\text{beat}}}\\ \;\mp 2r_{f_{0}f_{\text{beat}}}\sigma_{f_{0}}\sigma_{f_{\text{beat}}}-2r_{f_{0}f_{\text{rep}}}n\sigma_{f_{0}}\sigma_{f_{\text{rep}}}, \end{array}$$ (2)

where *σ_x_*^2^ is the variance of quantity *x*, *n* is the known comb line index (~5 × 10^6^), and each *r_xy_* is a correlation coefficient calculated between quantity *x* and quantity *y*. The contributions to the variance for the single longest run are given in Table 1. It is interesting to note that the uncertainty in *f*_rep_ is almost exactly cancelled by the correlation term between *f*_rep_ and *f*_beat_. We attribute this to variations in the Cs reference frequency. Any fluctuations in the repetition rate due to instabilities of the Cs reference are not recorded in the composite measurement of the He-Ne laser frequency because the synthesizers and the counters share the same time-base.

When the terms are appropriately added together using Eq. (2) and taking into account the correct sign for the He-Ne beat, the sum equals the variance of the measured *ν*_He-Ne_ within 0.2 %. It is clear from the table that the contribution to the variance due to the measurement of *f*_0_ does not significantly reduce the precision of the measurement.

**Fig. 3:**
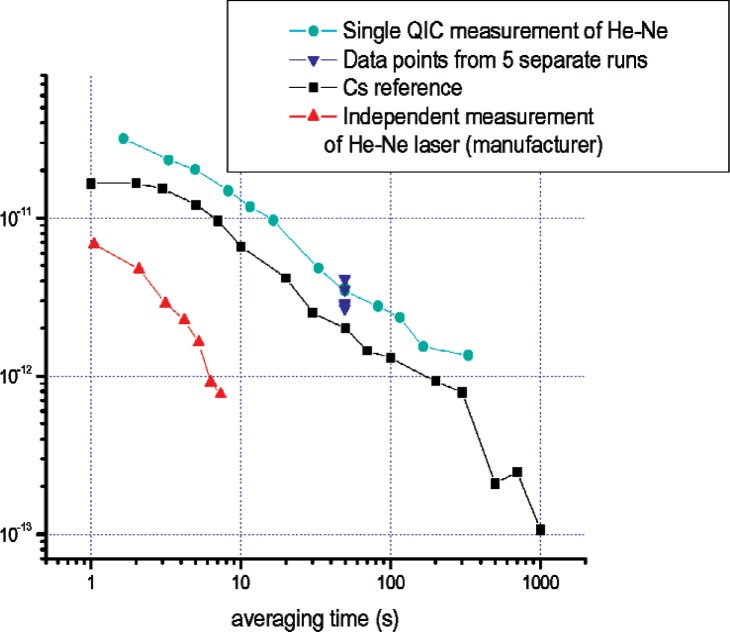
Allan deviation of the measured He-Ne laser frequency for a single measurement using QIC, a cesium primary standard (data taken April 2004 at JILA), and an independent measurement of the Allan deviation for the He-Ne laser frequency being measured [18]. The other points shown at 50 s averaging time represent the Allan deviation computed for 5 shorter runs taken the same day as the single QIC measurement.

We perform Allan deviation and correlation calculations on our data to understand the stability of our measurement for different averaging times. We account for a dead time of 0.65 s between one second measurements. The Allan deviation analysis of the measured He-Ne frequency reveals a trend with averaging time of *τ*^−1/2^, a signature of a white frequency-modulation noise spectrum. This is very similar in trend to the cesium reference time-base noise. For this type of noise, trivial corrections for dead time are necessary, as opposed to the use of more complex bias functions [20]. Fig. 3 gives the Allan deviation of the measured He-Ne frequency, as well as independently recorded Allan deviations for the He-Ne laser [15] and the cesium reference [21].

We correct for systematic shifts in the measured frequency because these are the only two conditions that necessitate a significant correction to our measured data. The output frequency depends on the pressure, which is related to the cold finger temperature in thermal equilibrium according to an empirical formula provided by the manufacturer. Pressure causes a collisional frequency shift of the iodine transition. We calculate that the correction due to the difference in temperature of the cell wall is −0.493 kHz. The systematic shift related to optical power is due to the AC Stark effect, which causes the hyperfine levels of the atoms to repel. The correction of +7.25 kHz is also added to correct for an intracavity power of 18.3 mW (compared with the CIPM recommended value of 10 mW). These two corrections were calculated from Taylor expansion coefficients provided by the manufacturer [22].

**Fig. 4:**
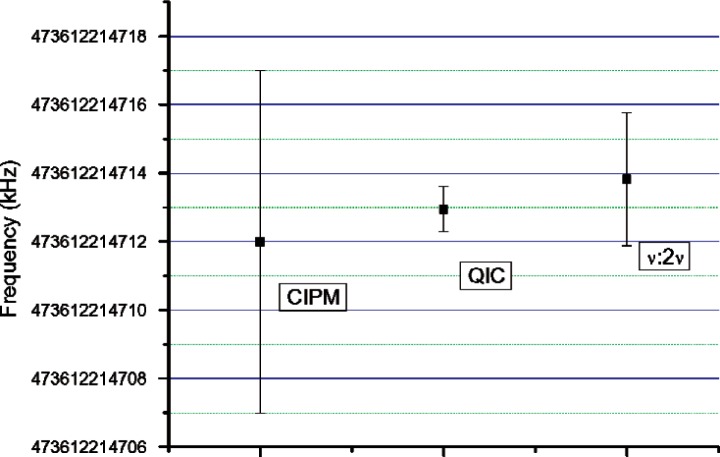
Comparison between QIC and *ν* - to - 2*ν* the standard self-referencing technique for optical comb metrology. Both results fall within the uncertainty of the CIPM recommended values. Allan deviations at the best averaging time in our measurements are 0.663 kHz for QIC, and 1.944 kHz for *ν* - to - 2*ν*.

Of the several data sets taken over the course of the day, the mean was 473 612 214 712.96 ± 0.663 kHz, which includes the two correctional shifts mentioned above. This value falls within the CIPM recommended range 473 612 214 712 ± 5 kHz. Fig. 4 gives a comparison of the results of our measurements using both the standard *ν* - to - 2*ν* technique and QIC for the locking of the carrier-envelope offset frequency with the CIPM standard. Although no improvement in the error can be conclusively attributed to the QIC method, the error may potentially be reduced if a device could be engineered to generate an octave spanning spectrum in the same semiconductor as the carrier-envelope offset frequency is detected, thus reducing the number of components in the setup [23].

## 4. Conclusion

We have used a femtosecond comb stabilized via quantum interference control to make an accurate and precise measurement of the frequency of an iodine-stabilized He-Ne laser. For averaging times of 330 s, the Allan deviation was 663 Hz. The frequency of the laser measured, after systematic correction shifts, was well within the CIPM recommended value for the ^127^I_2_ R(127) 11-5 i component. The fluctuations in the QIC measurement compare well with the fluctuations in the same measurement using the *ν* - to - 2*ν* technique.

## Figures and Tables

**Table 1:** Contributions to the variance from each term in Eq. (2) for the single longest run (~1100 data points). All values are in Hz^2^

$n^{2}\sigma_{f_{\text{rep}}}^{2}$	$\sigma_{f_{0}}^{2}$	$\sigma_{f_{\text{beat}}}^{2}$	$2r_{f_{\text{rep}}f_{\text{beat}}}n\sigma_{f_{\text{rep}}}\sigma_{f_{\text{rep}}}$	$2r_{f_{0}f_{\text{beat}}}\sigma_{f_{0}}\sigma_{f_{\text{beat}}}$	$2r_{f_{0}f_{\text{rep}}}\sigma_{f_{0}}\sigma_{f_{\text{rep}}}$
3.5 × 10^7^	2.3 × 10^−5^	2.3 × 10^8^	−3.4 × 10^7^	1.1 × 10^0^	1.6 × 10^1^

## Footnotes

1 Winters Electro-Optics Model 100 Iodine-stabilized He-Ne laser; mention of a brand name is for technical information only and does not represent an endorsement by NIST.

2 HP 5071A Primary frequency standard; mention of a brand name is for technical information only and does not represent an endorsement by NIST.
